# Supplementary material for: Assessment of organizational readiness to implement an electronic health record system in a low-resource settings cancer hospital: A cross-sectional survey
Source: PLoS One. 2020 Jun 16;15(6):e0234711. doi: 10.1371/journal.pone.0234711 (PMC7297346; doi:10.1371/journal.pone.0234711)
Supplement: S1 File — (DOCX) [file pone.0234711.s001.docx]

Questionnaire

*Note: 1. Model label column was not included in the questionnaire administered to participants.*

*2. Statements in Italics are negatively phrased and thus were reverse coded during analysis*

*3. We made five versions of the questionnaire with the 39 model items shuffled randomly*

**Questionnaire on organizational readiness to implement an electronic health records system**

Participant initials: _____________ Date: (DD/MM/YYYY) ________________

**Introduction**: This survey aims to assess your organization’s readiness to implement an electronic health records (EHR) system. For the purpose of this survey, an EHR system means any system of computer software and hardware used for collection, storage and sharing of patient information (such as patient identification, clinical history, diagnosis and treatments, lab and imaging results, etc) in a digital format as opposed to paper files. An example of such system is Clinic Master or OpenMRS. We want to get your personal opinion or perception of how ready you think your organization is to implement such systems. Your opinions will be valuable in informing us on how best to implement our EHR system, so we encourage you to carefully complete this survey, including detailed comments on the statements to explain your scores. There is no right or wrong answer, so freely express your opinion. The opinions you share in this survey will not be used in any way for assessing your work performance, and they will be analyzed as aggregate data without identifying individuals. Do not provide your name in this survey.

***Please tell us about you***

1. **Age** (Years) __________ 2. **Gender**: Male ☐ Female ☐

3. **How long have been working at this organization**? (Years): __________

4. **Highest education level**: *Please tick one that most applies to you*

High school ☐ Certificate ☐ Diploma ☐ Bachelors ☐ Masters ☐ PhD/Fellowship ☐

5. **Job title**: *Please tick one which best describes your job or role in this organization*

Oncologist ☐ Doctor ☐ Nurse ☐ Allied health worker (including lab tech) ☐ IT/Informatics ☐ Biostatistics/Data manager ☐ Administrator ☐

Support staff (e.g. store managers, secretary, etc) ☐

6. **Does your daily work involve direct patient care?**  Yes ☐ No ☐

7. **How often do you use a computer**? Daily ☐ A few times a week ☐ A few times a month ☐ A few times a year ☐ Never ☐

8. **How would you rate your computer skills on a scale of 1 to 5?** 1= Basic computer skills (need help with internet and email or office applications), 5= Proficient (able to do advanced tasks such as database management or programming)

Computer skills: *circle your level*


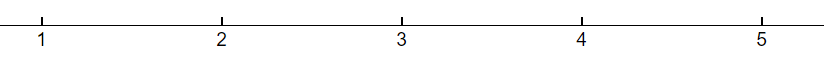
 Basic Proficient

9. **Do you have any experience using electronic medical records (EHR) systems?** Yes ☐ No ☐

10. **Have you ever received any training on EHRs?** Yes ☐ No ☐

Please score each of the following 39 statements on how well they describe your opinion about your organization with regards to implementing an electronic health records (EHR) system. Circle the number between 1 and 5 that represents your level of agreement or disagreement with each statement.

| **Statement** | **Strongly disagree** | **Disagree** | **Neither agree nor disagree** | **Agree** | **Strongly agree** | **Comment** | **Label in model** |
| --- | --- | --- | --- | --- | --- | --- | --- |
| I believe there are legitimate reasons for us to introduce a computer-based system in our unit. | 1 | 2 | 3 | 4 | 5 |  | VC1 |
| We definitely need new tools to improve the way we work around here. | 1 | 2 | 3 | 4 | 5 |  | VC2 |
| There are a number of rational reasons for the deployment of EHR system in our unit. | 1 | 2 | 3 | 4 | 5 |  | VC3 |
| A computer-based system is needed to improve our clinical processes. | 1 | 2 | 3 | 4 | 5 |  | VC4 |
| I think that staff in our unit will benefit from the use of an EHR. | 1 | 2 | 3 | 4 | 5 |  | CA1 |
| The deployment of an EHR will contribute to our unit's overall performance. | 1 | 2 | 3 | 4 | 5 |  | CA2 |
| The deployment of an EHR matches the priorities of our unit. | 1 | 2 | 3 | 4 | 5 |  | CA3 |
| The implementation of an EHR will prove to be best for our unit. | 1 | 2 | 3 | 4 | 5 |  | CA4 |
| I know staff outside our unit who had successful experiences with an EHR. | 1 | 2 | 3 | 4 | 5 |  | CE1 |
| An EHR has been successfully deployed in clinical units similar to ours. | 1 | 2 | 3 | 4 | 5 |  | CE2 |
| An EHR has received positive reviews in the press (e.g., newspapers, magazines, seminars, etc) | 1 | 2 | 3 | 4 | 5 |  | CE3 |
| I believe the government/ministry’s movement toward the electronic medical record represents a driving force for the deployment of an EHR in our unit | 1 | 2 | 3 | 4 | 5 |  | CE4 |
| Managers in our unit are committed to the deployment of an EHR. | 1 | 2 | 3 | 4 | 5 |  | TMS1 |
| Managers in our unit have stressed the importance of this change. | 1 | 2 | 3 | 4 | 5 |  | TMS2 |
| Managers have sent a clear message that the deployment of an EHR will occur in our unit. | 1 | 2 | 3 | 4 | 5 |  | TMS3 |
| Staff have been encouraged to embrace the upcoming deployment of an EHR. | 1 | 2 | 3 | 4 | 5 |  | TMS4 |
| There is a champion who actively promotes the deployment of an EHR in our unit. | 1 | 2 | 3 | 4 | 5 |  | C1 |
| The EHR project has a credible and trustworthy champion. | 1 | 2 | 3 | 4 | 5 |  | C2 |
| There is a champion who will be able to push the EHR project over or around implementation hurdles. | 1 | 2 | 3 | 4 | 5 |  | C3 |
| Our unit has successfully implemented other technological changes in recent years. | 1 | 2 | 3 | 4 | 5 |  | OHC1 |
| *Staff in our unit have had negative experiences with technological projects in the past.* | 1 | 2 | 3 | 4 | 5 |  | OHC2 |
| Our unit is usually successful when it undertakes all types of changes. | 1 | 2 | 3 | 4 | 5 |  | OHC3 |
| Information technology initiatives have been encouraged and are common practices in our unit. | 1 | 2 | 3 | 4 | 5 |  | OHC4 |
| Mutual trust and cooperation among staff in our unit is strong. | 1 | 2 | 3 | 4 | 5 |  | OCP1 |
| *Recent attempts to change the way we work in our unit have been hindered by political forces or conditions.* | 1 | 2 | 3 | 4 | 5 |  | OCP2 |
| *The climate in our unit is mainly characterized by conflicts and disputes.* | 1 | 2 | 3 | 4 | 5 |  | OCP3 |
| *Staff frustration is common in our unit.* | 1 | 2 | 3 | 4 | 5 |  | OCP4 |
| Our unit is structured to allow superiors to make changes quickly. | 1 | 2 | 3 | 4 | 5 |  | OF1 |
| It is easy to change procedures in our unit to meet new conditions. | 1 | 2 | 3 | 4 | 5 |  | OF2 |
| *Getting anything changed in our unit is a long, time-consuming process.* | 1 | 2 | 3 | 4 | 5 |  | OF3 |
| Policies and procedures in our unit allow us to take on new challenges effectively | 1 | 2 | 3 | 4 | 5 |  | OF4 |
| All staff in our unit are highly computer literate. | 1 | 2 | 3 | 4 | 5 |  | CSE1 |
| It won't take a long time before staff in our unit feel comfortable using an EHR. | 1 | 2 | 3 | 4 | 5 |  | CSE2 |
| Using a computer effectively is no problem for the staff in our unit. | 1 | 2 | 3 | 4 | 5 |  | CSE3 |
| *In general, staff in our unit have low computer skills.* | 1 | 2 | 3 | 4 | 5 |  | CSE4 |
| I believe an EHR can be successfully implemented in our unit. | 1 | 2 | 3 | 4 | 5 |  | OR1 |
| *Managers should delay the deployment of an EHR in our unit.* | 1 | 2 | 3 | 4 | 5 |  | OR2 |
| The deployment of an EHR in our unit is timely. | 1 | 2 | 3 | 4 | 5 |  | OR3 |
| Our unit is ready to take on this technological change. | 1 | 2 | 3 | 4 | 5 |  | OR4 |

Please give a general comment about your opinions. Why did you score the way you did? Why do you think your organization is ready or not ready to implement an EHR?

……………………………………………………………………………………………………………………………………………………………………........................................................................................................................................................................................................................................................................................................................................................................................................................................................................................................................................................................................................................................................................................................................................................................................................................................................................................................................................................................................................................................................................................................................................................................................................................................................................................................................................................
