# Supplementary material for: Assessment of organizational readiness to implement an electronic health record system in a low-resource settings cancer hospital: A cross-sectional survey
Source: PLoS One. 2020 Jun 16;15(6):e0234711. doi: 10.1371/journal.pone.0234711 (PMC7297346; doi:10.1371/journal.pone.0234711)
Supplement: S2 File — (DOCX) [file pone.0234711.s002.docx]

**Appendix 2: Description of SEM and PLS**

SEM (structural equation modeling) is group of multivariate statistical techniques that combine regression and factor analysis to examine complex, multi-level relationships between theory-based latent variables and their directly observed indicator variables (26–28). There are generally two approaches to SEM i.e. (i) PLS (partial least squares) which takes the ordinary least squares approach, and (ii) covariance-based SEM which takes the maximum likelihood estimation approach. PLS is suited for when there is co-linearity between the indicator variables or when there are many variables compared to the number of cases (26–28). In addition, PLS does not assume a normal distribution of the data, and is suitable for small sample sizes (27,29,30).

A PLS SEM model consists of two parts: (i) the measurement model (or outer model), which shows the relationship between the indicator variables and latent variables, and (ii) the structural model (or inner model), which shows the relationship within the latent variables. The structural model is the second level in which latent variables from the measurement model function as input (called exogenous latent variable) for other higher-level latent variables (called endogenous latent variable) (29).

The relationship between variables, shown by arrows in the path diagram (model), can be reflective or formative (29). In reflective models (called mode A in PLS SEM), the arrows run from the latent variables to the indicator variables, showing the assumption that the latent variable gives rise to, or explains, the indicator variable – i.e. the indicator variables are a manifestation of the underlying latent construct. The measurement part of our model (i.e. the relationship between the latent variables and the questionnaire items) falls in this type. In reflective measurement, the indicator variables are viewed as a representative sample of all possible items available within the conceptual domain of the latent variable they are measuring or as different ways of measuring the latent variable. Therefore the indicator variables should be interchangeable and any of the indicator variables can be left out without changing the latent variable. The indicator variables for a given latent variable are also highly correlated with each other since they are caused by the same latent variable. Conversely, in formative models (called mode B in PLS SEM) the arrows run from the indicator variables to the latent variables showing that the indicator variables cause or explain the latent variable. The latent variable is essentially a summary of the indicator variables taken jointly, with each indicator variable measuring a specific aspect of the latent variable. In these models, the indicator variables are independent and uncorrelated. The structural part of our model takes a formative type, with the endogenous latent variable (Organizational readiness, OR) as the outcome and all the other nine latent variables functioning as the indicators.

The R code for our analysis is provided along with the raw data in the additional files.
