# Supplementary material for: Assessment of organizational readiness to implement an electronic health record system in a low-resource settings cancer hospital: A cross-sectional survey
Source: PLoS One. 2020 Jun 16;15(6):e0234711. doi: 10.1371/journal.pone.0234711 (PMC7297346; doi:10.1371/journal.pone.0234711)
Supplement: S3 File — (DOCX) [file pone.0234711.s003.docx]

| Appendix 3: Cross-loadings. | | | | | | | | | | | |
| --- | --- | --- | --- | --- | --- | --- | --- | --- | --- | --- | --- |
| **Latent variable** | **Indicator variable** | **vc** | **ca** | **ce** | **tms** | **c** | **ohc** | **ocp** | **of** | **cse** | **or** |
| vc | vc1 | **0.69** | 0.33 | 0.27 | 0.30 | 0.23 | 0.26 | -0.07 | 0.13 | 0.12 | 0.34 |
|  | vc2 | **0.81** | 0.44 | 0.26 | 0.21 | 0.14 | 0.29 | -0.10 | 0.08 | 0.20 | 0.39 |
|  | vc3 | **0.70** | 0.47 | 0.38 | 0.21 | 0.29 | 0.18 | -0.16 | 0.08 | 0.11 | 0.34 |
|  | vc4 | **0.83** | 0.50 | 0.22 | 0.29 | 0.22 | 0.25 | 0.01 | 0.18 | 0.22 | 0.45 |
| ca | ca1 | 0.51 | **0.84** | 0.48 | 0.31 | 0.39 | 0.38 | 0.00 | 0.25 | 0.37 | 0.56 |
|  | ca2 | 0.60 | **0.79** | 0.33 | 0.23 | 0.28 | 0.34 | -0.06 | 0.20 | 0.20 | 0.48 |
|  | ca3 | 0.27 | **0.71** | 0.35 | 0.42 | 0.30 | 0.41 | 0.18 | 0.49 | 0.33 | 0.50 |
|  | ca4 | 0.49 | **0.86** | 0.42 | 0.37 | 0.38 | 0.40 | 0.11 | 0.30 | 0.39 | 0.62 |
| ce | ce1 | 0.25 | 0.37 | **0.60** | 0.27 | 0.32 | 0.15 | -0.11 | 0.14 | 0.16 | 0.26 |
|  | ce2 | 0.19 | 0.24 | **0.58** | 0.19 | 0.24 | 0.32 | 0.13 | 0.18 | 0.39 | 0.27 |
|  | ce3 | 0.27 | 0.38 | **0.63** | 0.28 | 0.26 | 0.29 | 0.09 | 0.05 | 0.07 | 0.22 |
|  | ce4 | 0.27 | 0.37 | **0.82** | 0.33 | 0.33 | 0.29 | 0.04 | 0.17 | 0.13 | 0.53 |
| tms | tms1 | 0.39 | 0.30 | 0.39 | **0.78** | 0.29 | 0.43 | 0.33 | 0.38 | 0.43 | 0.43 |
|  | tms2 | 0.33 | 0.35 | 0.29 | **0.76** | 0.33 | 0.38 | 0.22 | 0.37 | 0.52 | 0.45 |
|  | tms3 | 0.11 | 0.28 | 0.28 | **0.73** | 0.43 | 0.45 | 0.12 | 0.42 | 0.27 | 0.35 |
|  | tms4 | 0.16 | 0.35 | 0.27 | **0.79** | 0.38 | 0.44 | 0.28 | 0.39 | 0.38 | 0.42 |
| c | c1 | 0.25 | 0.37 | 0.40 | 0.39 | **0.85** | 0.28 | -0.10 | 0.14 | 0.24 | 0.44 |
|  | c2 | 0.27 | 0.39 | 0.32 | 0.36 | **0.80** | 0.25 | 0.06 | 0.20 | 0.19 | 0.39 |
|  | c3 | 0.14 | 0.24 | 0.28 | 0.34 | **0.72** | 0.30 | 0.09 | 0.05 | 0.22 | 0.30 |
| ohc | ohc1 | 0.25 | 0.32 | 0.23 | 0.37 | 0.31 | **0.59** | 0.08 | 0.24 | 0.23 | 0.23 |
|  | ohc2 | 0.14 | 0.08 | -0.02 | 0.01 | 0.11 | **0.19** | 0.16 | 0.00 | 0.14 | 0.13 |
|  | ohc3 | 0.20 | 0.37 | 0.20 | 0.34 | 0.18 | **0.73** | 0.19 | 0.35 | 0.26 | 0.42 |
|  | ohc4 | 0.25 | 0.36 | 0.42 | 0.52 | 0.30 | **0.84** | 0.30 | 0.35 | 0.49 | 0.47 |
| ocp | ocp1 | -0.05 | 0.14 | 0.15 | 0.32 | 0.13 | 0.32 | **0.86** | 0.37 | 0.27 | 0.20 |
|  | ocp2 | -0.11 | -0.06 | -0.16 | 0.09 | -0.16 | 0.12 | **0.58** | 0.28 | 0.05 | 0.09 |
|  | ocp3 | -0.08 | -0.02 | 0.00 | 0.14 | -0.12 | 0.05 | **0.57** | 0.14 | 0.14 | 0.06 |
|  | ocp4 | -0.02 | 0.00 | -0.02 | 0.17 | -0.01 | 0.19 | **0.43** | 0.20 | 0.24 | 0.03 |
| of | of1 | 0.15 | 0.33 | 0.35 | 0.47 | 0.27 | 0.37 | 0.29 | **0.74** | 0.25 | 0.44 |
|  | of2 | 0.11 | 0.29 | 0.04 | 0.41 | 0.09 | 0.26 | 0.26 | **0.75** | 0.41 | 0.40 |
|  | of3 | 0.03 | 0.11 | -0.10 | 0.06 | -0.07 | 0.13 | 0.36 | **0.47** | 0.24 | 0.20 |
|  | of4 | 0.10 | 0.26 | 0.15 | 0.33 | 0.08 | 0.37 | 0.30 | **0.75** | 0.38 | 0.34 |
| cse | cse1 | 0.08 | 0.30 | 0.30 | 0.39 | 0.36 | 0.42 | 0.21 | 0.35 | **0.79** | 0.43 |
|  | cse2 | 0.31 | 0.42 | 0.22 | 0.54 | 0.17 | 0.39 | 0.30 | 0.46 | **0.78** | 0.52 |
|  | cse3 | 0.13 | 0.23 | 0.14 | 0.33 | 0.14 | 0.31 | 0.11 | 0.28 | **0.78** | 0.39 |
|  | cse4 | 0.10 | 0.23 | 0.06 | 0.28 | 0.15 | 0.29 | 0.14 | 0.27 | **0.66** | 0.28 |
| or | or1 | 0.54 | 0.49 | 0.33 | 0.37 | 0.29 | 0.40 | 0.18 | 0.39 | 0.40 | **0.73** |
|  | or2 | 0.30 | 0.48 | 0.21 | 0.12 | 0.14 | 0.19 | -0.11 | 0.16 | 0.17 | **0.53** |
|  | or3 | 0.28 | 0.42 | 0.46 | 0.24 | 0.30 | 0.27 | -0.03 | 0.27 | 0.27 | **0.63** |
|  | or4 | 0.28 | 0.53 | 0.45 | 0.60 | 0.50 | 0.53 | 0.31 | 0.51 | 0.57 | **0.84** |
