# Supplementary material for: Assessment of organizational readiness to implement an electronic health record system in a low-resource settings cancer hospital: A cross-sectional survey
Source: PLoS One. 2020 Jun 16;15(6):e0234711. doi: 10.1371/journal.pone.0234711 (PMC7297346; doi:10.1371/journal.pone.0234711)
Supplement: Attachment — Submitted filename: Response to Reviewers.docx [file pone.0234711.s007.docx]

Review Comments to the Author

Reviewer #1

The authors have addressed all comments. But I think the title could benefit from a reword. It is lengthy. Kindly replace "structural equation modeling of survey data to identify relevant variables" with "a cross-sectional survey"

Response: Thank you for this suggestion. We have revised the title, and now it reads: “Assessment of organizational readiness to implement an electronic health record system in a low-resource settings cancer hospital: a cross-sectional survey”
